# The intriguing dose-dependent effect of selected amphiphilic compounds on insulin amyloid aggregation: Focus on a cholesterol-based detergent, Chobimalt

**DOI:** 10.3389/fmolb.2022.955282

**Published:** 2022-08-19

**Authors:** Katarina Siposova, Viktor I. Petrenko, Ivana Garcarova, Dagmar Sedlakova, László Almásy, Olena A. Kyzyma, Manfred Kriechbaum, Andrey Musatov

**Affiliations:** ^1^ Department of Biophysics, Institute of Experimental Physics, Slovak Academy of Sciences, Kosice, Slovakia; ^2^ BCMaterials—Basque Center for Materials, Applications and Nanostructures, Leioa, Spain; ^3^ Ikerbasque, Basque Foundation for Science, Bilbao, Spain; ^4^ Neutron Spectroscopy Department, Centre for Energy Research, Budapest, Hungary; ^5^ Faculty of Physics, Taras Shevchenko National University of Kyiv, Kyiv, Ukraine; ^6^ Institute of Inorganic Chemistry, Graz University of Technology, Graz, Austria

**Keywords:** Chobimalt, cholesterol-based, detergent, amphiphile, insulin, amyloid aggregation, fibrillar morphology

## Abstract

The amyloidogenic self-assembly of many peptides and proteins largely depends on external conditions. Among amyloid-prone proteins, insulin attracts attention because of its physiological and therapeutic importance. In the present work, the amyloid aggregation of insulin is studied in the presence of cholesterol-based detergent, Chobimalt. The strategy to elucidate the Chobimalt-induced effect on insulin fibrillogenesis is based on performing the concentration- and time-dependent analysis using a combination of different experimental techniques, such as ThT fluorescence assay, CD, AFM, SANS, and SAXS. While at the lowest Chobimalt concentration (0.1 µM; insulin to Chobimalt molar ratio of 1:0.004) the formation of insulin fibrils was not affected, the gradual increase of Chobimalt concentration (up to 100 µM; molar ratio of 1:4) led to a significant increase in ThT fluorescence, and the maximal ThT fluorescence was 3-4-fold higher than the control insulin fibril’s ThT fluorescence intensity. Kinetic studies confirm the dose-dependent experimental results. Depending on the concentration of Chobimalt, either (i) no effect is observed, or (ii) significantly, ∼10-times prolonged lag-phases accompanied by the substantial, ∼ 3-fold higher relative ThT fluorescence intensities at the steady-state phase are recorded. In addition, at certain concentrations of Chobimalt, changes in the elongation-phase are noticed. An increase in the Chobimalt concentrations also triggers the formation of insulin fibrils with sharply altered morphological appearance. The fibrils appear to be more flexible and wavy-like with a tendency to form circles. SANS and SAXS data also revealed the morphology changes of amyloid fibrils in the presence of Chobimalt. Amyloid aggregation requires the formation of unfolded intermediates, which subsequently generate amyloidogenic nuclei. We hypothesize that the different morphology of the formed insulin fibrils is the result of the gradual binding of Chobimalt to different binding sites on unfolded insulin. A similar explanation and the existence of such binding sites with different binding energies was shown previously for the nonionic detergent. Thus, the data also emphasize the importance of a protein partially-unfolded state which undergoes the process of fibrils formation; i.e., certain experimental conditions or the presence of additives may dramatically change not only kinetics but also the morphology of fibrillar aggregates.

## 1 Introduction

Loss of protein homeostasis is a common feature of aging and diseases that are characterized by the appearance of nonnative protein aggregates in various tissues (Powers et al., 2009; Labbadia, and Morimoto, 2015; Lévy et al., 2019). Although the proteins causing amyloid diseases show sequence, size, and function diversity, they all form similar amyloid fibrils consisting of the same cross-β structure, i.e., β-strands arranged perpendicular to the long fibril axis. However, proteins differ significantly in their propensity and the conditions in which they form fibrils (Pellarin and Caflish, 2006; Knowles et al., 2007; Krebs et al., 2007; Rambaran and Serpell, 2008; Zhuravlev et al., 2014; Bernson, et al., 2020). Among amyloidogenic proteins, insulin attracts attention because of its physiological and therapeutic importance. As the hormone, insulin regulates important cellular processes. Insulin significance lies not only in carbohydrate metabolism by facilitating glucose diffusion into fat and muscle cells, but in addition, insulin plays a key role in the synthesis and storage of fatty acids, phospholipid metabolism, urea cycle, and is involved in cell proliferation (Straus, 1981; Hunter and Garvey, 1998; Wilcox, 2005). A growing body of epidemiological and molecular evidence now suggests an obvious relationship between brain insulin resistance, dementia due to Alzheimer's disease (AD), Parkinson's disease (PD), and AD/PD-related dementias (Bosco et al., 2012; de la Monte, 2012; Talbot, 2014; Athauda and Foltynie, 2016; Biessels and Despa, 2018; Ferreira et al., 2018; Hong et al., 2020). On the other hand, subcutaneous applications of insulin during the treatment of diabetes may cause insulin-derived amyloidosis characterized by the formation of insulin amyloid fibrils at the site of repeated injections (Dische et al., 1988; D'Souza et al., 2014; Nagase et al., 2014; Nilsson, 2016). In addition, the presence of insulin oligomers/fibrils may induce autoimmune responses as documented for Parkinson´s patients, which suggests the involvement of insulin in Parkinson's pathogenesis (Wilhelm, et al., 2007). However, it should be noted that the aggregation of insulin has only been observed with externally administered forms of insulin, and not when it is normally produced in the human body (Das et al., 2022). *In vitro* insulin can be easily transferred into the amyloid state, if appropriate, typically denaturing conditions are applied (Sluzky et al., 1991; Brange et al., 1997; Nielsen et al., 2001; Malik and Roy, 2011). Undesirable insulin aggregation is one of the major issues in biopharmaceutical production, storage, and insulin amyloid aggregates have been observed in insulin pumps or insulin infusion systems (Brange and Langkjoer, 1993; Brunetti, et al., 2003; Guilhem et al., 2006).

Understanding how amyloid fibrillization can be modulated is undoubtedly very important when studying amyloid aggregation. Particularly important appears to be the role of hydrophobic interaction of amyloid-prone proteins/peptides with membranes and/or with the individual membrane components (Jelinek and Sheynis, 2010; Nault et al., 2013). It is currently accepted, that membrane composition is the factor controlling the aggregation process (Necula et al., 2003; Gorbenko and Kinnunen, 2006; Bucciantini et al., 2014; Banerjee et al., 2021; Hashemi et al., 2022). Lipid bilayers may act as conformational catalysts, favoring protein misfolding and inducing the growth of aggregation nuclei, early oligomers, and mature fibrils with specific biophysical, structural, and toxicity features (Bucciantini et al., 2014; Korshavn et al., 2017; Rangachari et al., 2018). In fact, one of the hypotheses is that the hydrophobic interaction of amyloid aggregates with cell membranes resulted in the toxicity of amyloids (Jelinek and Sheynis, 2010; Mrdenovic et al., 2022).

A significant effect of lipid bilayers has been investigated in detail for Aβ peptide aggregation (Matsuzaki, 2007; Lin et al., 2009; Korshavn et al., 2017; Iadanza et al., 2018; Hashemi et al., 2022; Mrdenovic et al., 2022). The strongest evidence causally linking cholesterol to AD is provided by experimental studies showing that adding or reducing cholesterol alters amyloid precursor protein (APP) and Aβ peptide levels (McLaurin et al., 2003; Wood et al., 2003; Lin et al., 2009; Banerjee et al., 2021; Hashemi et al., 2022). For example, accumulation of cholesterol in membranes is associated with AD development, suggesting that insertion of cholesterol into membranes may initiate the Aβ aggregation, and significantly enhances the aggregation kinetics of Aβ (Banerjee et al., 2021; Hashemi et al., 2022). The enhanced aggregation propensity in the presence of cholesterol has also been observed for IAPP protein (Christensen et al., 2021).

The importance of membrane surface/composition is also observed for insulin aggregation, especially when composition differs from the native pancreatic β-cell’s membrane, where insulin is secreted. It appears that a significant role is played by cholesterol, which is typically absent in pancreatic β-cells. Previously, using two model membranes, namely large unilamellar vesicles (LUVs) consisting of DOPC and DOPS which mimic the pancreatic β-cell membrane, and LUVs composed of POPC and cholesterol which mimic the eukaryotic cell membranes it was demonstrated, that the presence of cholesterol led to the accelerated aggregation of insulin (Engel et al., 2008; Ratha et al., 2018). Cholesterol plays a central role in the structural and functional integrity of cellular membranes. Interaction of cholesterol with proteins including amyloid-prone proteins can alter their stability and function. There is a large body of evidence suggesting that cholesterol homeostasis in the brain is linked to AD. These findings account for the increasing interest to study the interaction of amyloid-prone proteins with cholesterol. However, cholesterol and most cholesterol derivatives have poor solubility, and therefore, do not permit the use of some of the experimental techniques in vitro. This issue can be overcome by using a well soluble in water cholesterol-based detergent, Chobimalt, in which cholesterol is present as a hydrophobic building block (Howell et al., 2010, Figure 1).

**FIGURE 1:**
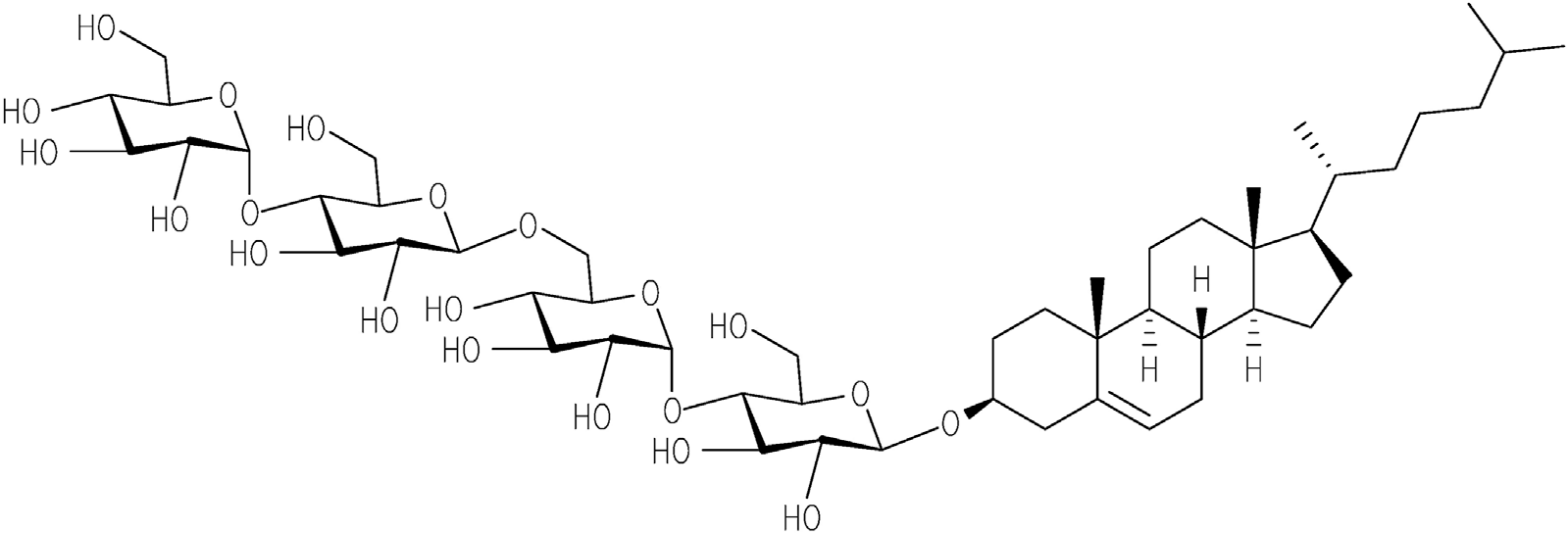
Chemical structure of Chobimalt (Howell et al., 2010).

It has been demonstrated that Chobimalt forms large micelles at concentrations above 3–4 µM (critical micelle concentration), therefore the detergent can be used as a model of a cholesterol-based artificial membrane system (Howell et al., 2010). Detergents are often used to understand the biophysical and biochemical consequences of the protein aggregation process under conditions that mimic physiological conditions, i.e., the surface of a biological membrane. In addition, since detergents are amphiphilic compounds, they can provide valuable information about hydrophobic interactions that play an important role in the formation of amyloid fibrils (Sharp et al., 2002; Kayed et al., 2004; Moores et al., 2011; Ryan et al., 2012; Abelein et al., 2013; Österlund et al., 2018). Previous studies have indicated that the modulation effect of surfactants on amyloid fibrillation can be multifaceted and it is highly dependent on the actual amyloidogenic protein systems and fibrillization conditions (Giehm et al., 2010; Hung et al., 2010; Otzen, 2010; Khan, et al., 2012; Khan et al., 2014; Siposova et al., 2019a; Siposova et al., 2021a).

Previously, we have successfully applied small-angle neutron scattering (SANS) and small-angle X-ray scattering (SAXS) techniques for structural investigations of insulin and lysozyme amyloid aggregates alone and within the complex and multicomponent protein-nanoparticles system (Avdeev et al., 2013; Majorosova et al., 2016; Siposova et al., 2020). Unlike AFM, which is used for the analysis of dried dilute samples, the SANS and SAXS methods allow studying structural/morphological changes of concentrated objects (proteins, amyloid aggregates) in an aqueous solution. In this work, we have used classical methods, such as Thioflavin T (ThT) fluorescence assay and atomic force microscopy (AFM), in combination with SANS and SAXS to investigate the effect of Chobimalt on insulin amyloid aggregation. In addition, the stability of the systems over time was investigated by repeating SANS measurements over a time interval of several days.

## 2 Materials and methods

### 2.1 Insulin amyloid fibrillization in presence of Chobimalt

To elucidate the effect of Chobimalt on insulin amyloid fibrillization, two experimental approaches have been used: i) evaluation of concentration/dose-dependent effect of Chobimalt on insulin fibrils formation, and ii) investigation of the Chobimalt effect on the kinetics of insulin fibrillization (time-dependent measurements). The formation of insulin amyloid fibrils was monitored by Thioflavin T fluorescence assay (ThT, T3516; Sigma-Aldrich, Inc., St. Louis, MO) and confirmed by atomic force microscopy (AFM).

Within both, dose- and time-dependent measurements, insulin (human recombinant, expressed in yeast, I2643; Sigma-Aldrich) was dissolved in 100 mM NaCl solution, pH 1.6 (hereinafter referred to as NaCl solution), to a final concentration of 25 μM and the solution was incubated in an Eppendorf comfort thermomixer at 65°C for 6 h under constant agitation (500 rpm). For dose-dependent experiments, insulin was incubated in the presence of increasing from 0.1 µM up to 1 mM Chobimalt concentrations, which correspond to insulin to Chobimalt molar ratio ranging from 1:0.004 to 1:40 (samples were incubated at 65°C, 500 rpm for 6 h). A stock solution of Chobimalt was prepared by dissolving Chobimalt powder in ultra-pure H_2_O. Chobimalt was added to insulin from freshly prepared Chobimalt stock solutions to reach final concentrations between 0.1 µM and 1 mM. For quantification of insulin fibrillization alone and in presence of Chobimalt, ThT fluorescence measurements were performed (*please see the section below*). For kinetic measurements, the aliquots of insulin solution incubated at 65°C in the absence and presence of the desired concentration of Chobimalt were withdrawn at varying times, mixed with ThT, incubated at 37°C for 1 h, and analyzed as described below.

### 2.2 Thioflavin T fluorescence assay

For quantification of insulin fibrillization alone and in presence of Chobimalt, ThT fluorescence measurements were performed. After the process of fibrillization, ThT was added to each sample to reach the final concentration ratio of insulin to ThT 1:5 (5 μM of insulin and a final ThT concentration of 25 μM) followed by incubation for 1 h at 37°C. The fluorescence intensity was measured using a 96-well plate by a Synergy MX (BioTek) spectrofluorometer. The excitation was set at 440 nm and the emission was recorded at 485 nm. The excitation and emission slits were adjusted to 9.0/9.0 nm and the top probe vertical offset was 6 nm. The fluorescence intensities of samples were normalized to the fluorescence intensities of amyloid insulin aggregates prepared in the absence of Chobimalt (taken as 100%). Each experiment was performed in triplicates; the error bars represent the average deviation for repeated measurements of three separate samples.

The experimental kinetic data were fitted by 4 parameters sigmoidal logistic equation using SigmaPlot version 14.0 (Systat Software Inc.) as described previously (Siposova et al., 2019a; Siposova et al., 2019b).

### 2.3 Circular dichroism spectroscopy

The far-UV circular dichroism (CD) spectra of native insulin (25 µM) in the absence and presence of a different concentration of Chobimalt and insulin fibrils formed in the absence and presence of Chobimalt were measured on a Jasco 815 CD spectropolarimeter (Jasco, Tokyo, Japan) over a wavelength range of 190–270 nm with a scan rate of 100 nm/min using a quartz cuvette with 0.1 cm path at 25 ± 1°C. The spectra represent the average of the three measurements obtained in acidic conditions, after subtracting the solvent background. Spectra of native insulin were taken immediately after the addition of Chobimalt to insulin solutions. Spectra of insulin amyloid fibrils formed in the absence and presence of Chobimalt were taken after fibrillization (6 h).

### 2.4 Atomic force microscopy

Atomic force microscopy (AFM) was used for analyzing the morphology of formed insulin amyloid aggregates (*control*) and in the presence of Chobimalt. Samples for AFM were prepared by casting 10 μL aliquots on a freshly cleaved mica surface (the highest grade V1 mica discs, Ted Pella, Inc., Redding, CA). Using AFM, insulin fibrils formed in the absence (*control*) and insulin fibrils formed in the presence of a different concentration of Chobimalt have been analyzed. Aliquots were withdrawn after 6 h fibrillization and diluted 20x and 10x. After adsorption to the surface (5–10 min at 25°C), the mica surface was washed with ultrapure water (18.2 MΩ cm) and the samples were dried under a stream of nitrogen. The AFM images were obtained using a Scanning Probe Microscope (Veeco di Innova, Bruker AXS Inc., Madison, United States) working in a tapping mode. The scan rate was 0.5–0.75 kHz. The resolution of the image was 1024×1024 pixels/image). The AFM images were analyzed using NanoScope Analysis1.20 (Veeco di Innova, Bruker AXS Inc., Madison, WI, United States), and no smoothing or noise reduction was applied.

### 2.5 Small-angle scattering measurements

#### 2.5.1 Small-angle neutron scattering

SANS experiments were carried out using the Yellow Submarine diffractometer operating at the Budapest Neutron Center, Hungary (Almasy, 2021). Samples were placed in 2 mm-thick Hellma quartz cells. The temperature was set at 20°C and controlled within 0.1°C using a Julabo FP50 water circulation thermostat. The range of scattering vectors *q* was set to 0.07÷3.1 nm^-1^. The *q* value is defined as *q*=4 π⁄λ sinθ where 2θ is the scattering angle. To have access to the whole range of *q*, we used two different configurations with sample-detector distances of 1.15 and 5.125 m and mean neutron wavelengths of 0.6 and 1.04 nm. The raw data have been corrected for sample transmission, scattering from the empty cell, and room background. Correction of the detector efficiency and conversion of the measured scattering to an absolute scale was performed by normalization to scattering from the water.

#### 2.5.2 Small-angle X-ray scattering

SAXS measurements were carried out using a SAXSpoint 2.0 instrument (Anton Paar, Austria). Using Cu Kα radiation and a hybrid photon-counting 2D EIGER R series detector, a *q-*range of 0.07–5 nm^−1^ was covered with *q*-resolution δ*q* < 0.003 nm^−1^. The measurements were carried out on samples in solution at room temperature using a special quartz capillary of 1 mm in diameter.

For SANS/SAXS measurements, insulin amyloid fibrils were prepared by dissolving proteins in D_2_O−DCl, pD ∼ 2.0 to reach the insulin final concentration of 2 mg/mL (which corresponds to 344 µM). The samples containing Chobimalt were prepared to maintain the discrete protein to Chobimalt molar ratios.

## 3 Results and discussion

### 3.1 Concentration- and time-dependent effect of Chobimalt on insulin fibrillogenesis

The strategy to elucidate the Chobimalt-induced effect on insulin fibrillogenesis was based on performing the concentration- and time-dependent analysis. The formation of amyloid structures of insulin was controlled by a fluorescent analysis of thioflavin T (ThT) as the most common method based on the general agreement that the presence of fibrils leads to an increase in the intensity of ThT fluorescence and, conversely, with a decrease in the content of fibrils in the sample, the intensity of fluorescence decreases (LeVine and , 1993; Groenning et al., 2007; Vus et al., 2015; Sulatsky et al., 2020).

**FIGURE 2:**
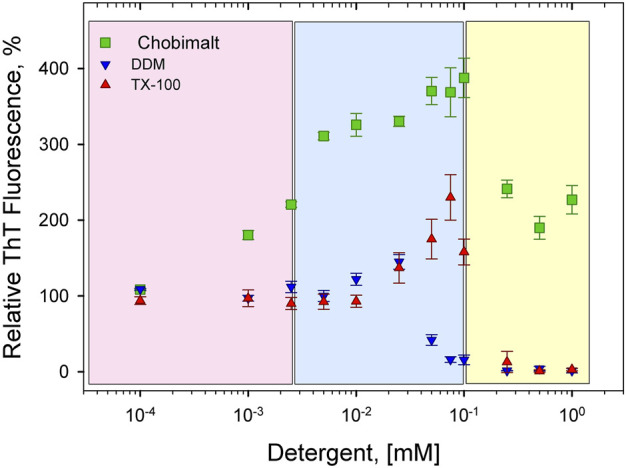
Concentration-dependent effect of Chobimalt on insulin fibrillization monitored by ThT fluorescence assay. The effect of Chobimalt on insulin fibrils formation was quantified as a function of Chobimalt concentration ranging from 0.1 µM to 1 mM at a fixed 25 µM concentration of insulin. Three colored areas represent a different fibrillization behavior as a function of Chobimalt concentration.

The concentration- and time-dependent experiments have been performed at fixed, 25 µM insulin concentration (dissolved in 100 mM NaCl, pH 1.6). Figure 2 shows a relative ThT fluorescence intensities of insulin incubated in the presence of increasing from 0.1 µM up to 1 mM Chobimalt concentrations, which correspond to insulin to Chobimalt molar ratio ranging from 1:0.004 to 1:40. At the lowest, 0.1 µM Chobimalt concentration, the formation of insulin fibrils was not affected and ThT fluorescence was similar to the control insulin samples (ThT fluorescence taken as 100%). Unexpectedly, even an initial increase in Chobimalt concentration up to 1 µM (ratio 1:0.04) caused an almost two-fold increase in ThT fluorescence. Gradual increase of concentration of Chobimalt up to 0.1 mM (up to 1:4 molar ratio) led to a further significant increase of ThT fluorescence and the maximal ThT was 3.5–3.9 times higher than control insulin fibril’s ThT fluorescence value. An additional increase in the concentration of Chobimalt did not lead to an increase in ThT fluorescence, but a decrease in ThT fluorescence was observed. Although a decrease of ThT occurred, the ThT fluorescence values remained ∼2 times higher than insulin alone, i.e., inhibition of insulin amyloid formation was not observed (Figure 2). Differently colored areas inset Figure 2 will be discussed later. Previously, we have demonstrated a similar spike of ThT fluorescence for insulin fibrils in the presence of non-ionic detergents, Triton X-100 (TX-100), dodecyl-maltoside (DDM), and detergent-phospholipid micelles (Siposova et al., 2019a; Siposova et al., 2021a). The molecular modeling suggested that a spike was caused by the successive occupation of the insulin binding sites by detergent monomers leading to subsequent morphological changes in generated fibrils (Siposova et al., 2019a; Siposova et al., 2021a). However, in the case of non-ionic detergents, at a high detergent to insulin ratio an inhibition of insulin fibrillation was detected (Siposova et al., 2019a; Siposova et al., 2021a). The different anti-amyloidogenic effects of non-ionic detergents (TX-100 and DDM) and Chobimalt can be explained by the different chemical structures which most likely affect their binding. In fact, DDM is a sugar-based, TX-100 is a surfactant with hydrophilic polyethylene oxide, while Chobimalt is a cholesterol-based detergent. It should be noted, that previously published data were obtained using 10 µM insulin. In the current work, the experiments were repeated with 25 µM of insulin and in the presence of different concentrations of DDM and TX-100 (Figure 2, blue and red triangles, respectively).

The obtained results fully support our previous observations and confirmed our previous conclusion that the ratio between the protein and detergent concentrations defines the outcome of the experiments (Siposova et al., 2019a; Siposova et al., 2021a). The modulation effect of surfactants on amyloid fibrillation can be multifaceted and it is highly dependent on the actual amyloidogenic protein systems and fibrillization conditions as observed previously. For example, a biphasic effect on lysozyme amyloid aggregation was revealed for sodium dodecyl sulfate (SDS) (Hung et al., 2010). In addition, it was shown that under controlled experimental conditions, especially pH, SDS can effectively induce amyloidogenesis in at least 25 proteins, in which a protonation seems to be crucial in SDS-induced amyloid fibrillization, usually via a micelle-surface-mediated mechanism (Giehm et al., 2010; Otzen, 2010; Khan et al., 2012; Khan et al., 2014). The “dual” mode of action was described for the seven-residue B-chain segment LVEALYL that can either delay or accelerate insulin fibril formation in a molar ratio-dependent manner (Ivanova et al., 2009). In equimolar ratios with insulin, LVEALYL inhibits fibrillation, and in much lower concentrations than insulin, LVEALYL accelerates it. This observation of B-chain segment LVEALYL on insulin fibril formation also suggests that this segment is important for the formation of the spine of the insulin fibrils (Ivanova et al., 2009).

**FIGURE 3:**
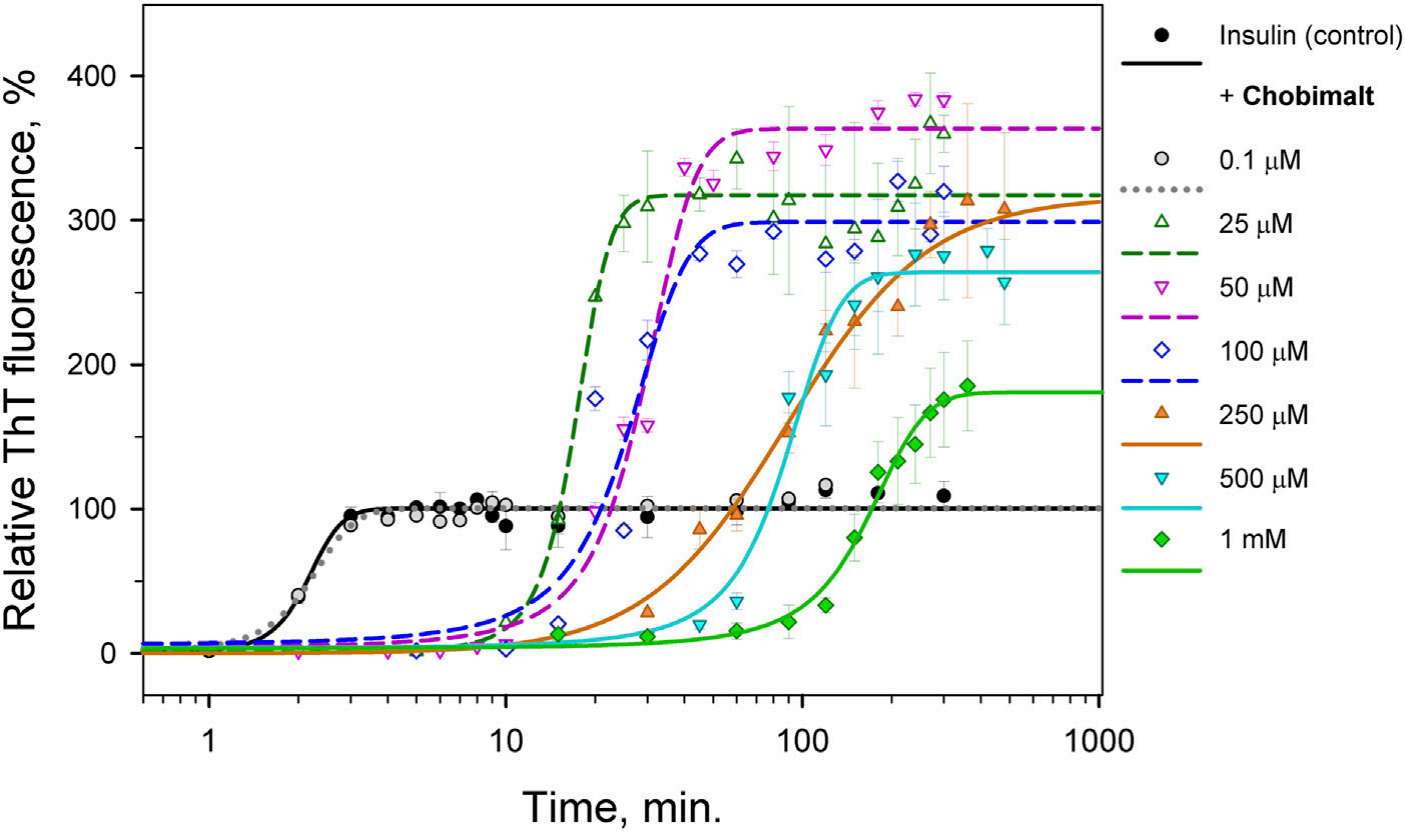
Time-dependence of insulin fibrillization in the presence of Chobimalt evaluated by ThT assay. Chobimalt has been added to freshly prepared insulin solution and samples were exposed to fibrillization conditions.

The effect of Chobimalt on the kinetics of insulin fibrillization is illustrated in Figure 3. The study of kinetics has been performed to *i*) obtain information about the time frame of fibrillization and *ii*) confirm the assumption of the importance of the insulin to Chobimalt ratio on insulin fibrillogenesis. In the absence of Chobimalt, the insulin kinetic curve exhibits a typical sigmoidal pattern with three, well-defined phases: short (∼1–2 min) lag phase, followed by a fast elongation phase, and finally, the curve approached a plateau phase, and reached a constant value after ∼3–4 min. This observation fully correlates with our previous detailed study of insulin fibrillization under similar conditions ( Siposova et al., 2021b). In the presence of 0.1 µM of Chobimalt, the extent and the kinetic of insulin fibrillization remained unaffected as documented by grey symbols and dotted line. However, when incubated in the presence of Chobimalt in the range of ∼25–100 µM, the insulin fibrillization showed a prolonged lag phase (up to 10–15 min) and the plateau phase was reached after 30 min in the presence of 25 µM Chobimalt, and after ∼40 min in the presence of 50 and 100 µM Chobimalt (*empty symbols fitted by short-dashed curves*). Similar to data illustrated in Figure 2, the relative ThT fluorescence intensities at the plateau phase was more than 3-fold higher than the control data. The presence of 250 and 500 µM Chobimalt led to a significantly prolonged lag phase and elongation phase start after ∼30–40 min of incubation. The most delayed lag phase was obtained in the presence of 1 mM Chobimalt. It should be mentioned that in the presence of Chobimalt at a concentration ≥ 25 µM, the elongation phase began at the time when fibrillization of the control insulin samples has already culminated.

**FIGURE 4:**
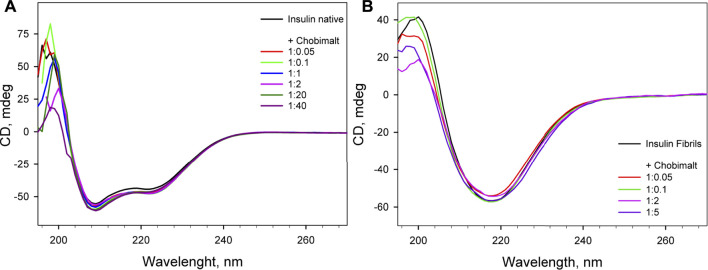
Far UV- circular dichroism spectra of native insulin in the absence and presence of different concentrations of Chobimalt **(A)**, and insulin amyloid fibrils formed in the absence and presence of different concentrations of Chobimalt **(B)**. Concentration of insulin was always 25 µM. The spectra were taken immediately after the detergent was added to protein **(A)** and after of the fibrillization process **(B)**.

CD spectroscopy was carried out to determine whether Chobimalt can alter the native structure of insulin and to verify whether insulin forms amyloid aggregates under selected experimental conditions. The CD spectrum in the far-UV region of the native insulin, at pH 1.6 before exposure to high temperature and agitation, exhibited negative bands at 222 and 208 nm which indicate the presence of an α-helical structure (Figure 4). When different concentrations of Chobimalt were added to native insulin, the observed spectra were almost identical to the spectra measured for insulin in the absence of Chobimalt, suggesting that Chobimalt does not alter the native insulin structure. However, insulin fibrils formed in the absence and presence of increased concentrations of Chobimalt show significant changes in the CD spectrum with a negative band at ∼218 nm, which corresponds to the presence of β-structural motif (typical cross β-structure of fibrils).

### 3.2 Atomic force microscopy visualization of fibrils

**FIGURE 5:**
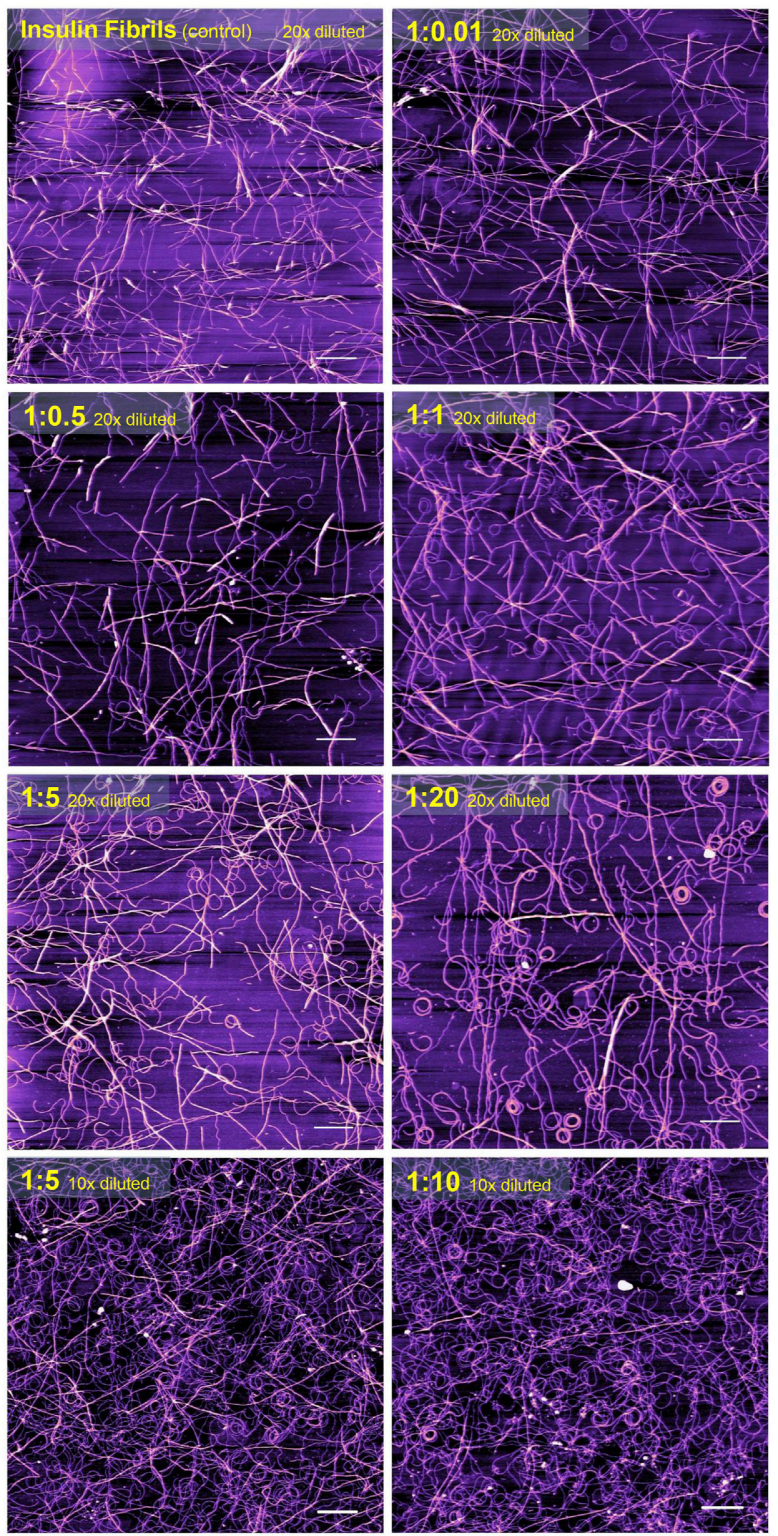
The atomic force microscopy visualization of insulin amyloid fibrils formed alone (*control*) and in the presence of different concentrations of Chobimalt. White scale bars always represent 1 µm. The images were processed using NanoScope Analysis 1.20 software.

It is generally recognized that *in vitro* fibrillization of insulin is highly dependent on several environmental factors, such as acidic or basic conditions, elevated temperature, ionic strength, agitation, presence of salts, and many others (Waugh, 1946; Sluzky et al., 1991; Nielsen, et al., 2001). The presence of these factors causes a partial, but very important unfolding of insulin, which is a key step at the beginning of the formation of the amyloidogenic nucleus (Hua and Weiss, 2004; Hong and Fink, 2005; Akbarian et al., 2018; Akbarian et al., 2020). We assume that the interaction of insulin molecules with Chobimalt leads to the formation of partially unfolded insulin molecules of various configurations, which subsequently affects the kinetic of insulin fibrillization and the morphology of resulting fibrils. It was already shown that at the amyloidogenic conditions causing partial unfolding of insulin, at least two major populations of partially unfolded intermediates are present, in which all three disulfide bonds remain intact (Ahmad et al., 2005; Zako, et al., 2009; Kurouski et al., 2012). The differences between these intermediates cause various fibrillization pathways that might generate morphologically different fibrils, which represent the basis of the amyloid fibril polymorphisms and the morphology of fibrils subsequently affects also the ThT fluorescence (Dzwolak et al., 2004; Stsiapura et al., 2008; Chatani et al., 2015; Sneideris et al., 2015; Eisenberg, and Sawaya, 2017). We demonstrated that the propensity of insulin to form fibrils is affected by Chobimalt in a concentration- and time-dependent manner (Figure 2, 3). We observed a significantly changed both, lag phase and extension of fibrillization as a function of insulin to Chobimalt ratio. To elucidate the effect of Chobimalt on the morphology of formed amyloid fibrils, AFM has been performed. Figure 5 represents AFM scans of insulin fibrils formed alone and in the presence of different concentrations of Chobimalt, as indicated by the molar ratios. The control insulin fibrils display typical morphology, i.e. thin, long, unbranched insulin fibrils were observed. In the presence of a low, 0.25 µM concentration of Chobimalt (ratio 1:0.01) the morphology of fibrils remained almost the same; and no visible changes were observed. However, a further increase in Chobimalt concentration triggered the formation of insulin fibrils with a significantly changed morphological appearance. As documented, at ratios ≥1:0.5, the fibrils appear to be more flexible, wavy fibrils with the tendency to form circles. Even more pronounced wavy- and curved fibrils arranged in circles we observed at insulin to Chobimalt ratios higher than 1:5 as presented in Figure 5. The results obtained using a combination of dose- and time-dependent measurements, and AFM suggest that the effect of Chobimalt on insulin fibrillization can be divided, at least hypothetically, into three stages. Although at the first stage, i.e., at a low concentration of Chobimalt (pink area in Figure 2), a small effect on the extent of fluorescence intensity was observed, no effect was found on either, kinetics or fibrils morphology. In the second stage, the increased concentration of Chobimalt (up to 100 µM; ratio 1:4) led to a significant increase in ThT fluorescence (blue area in Figure 2), a prolonged lag-phase of fibrillization (Figure 3), and a steady-state phase that occurred after ∼40 min. In addition, AFM scans revealed the formation of flexible and wavy fibrils. The last, third stage is observed at the highest Chobimalt concentration (up to 1 mM) and protein to the detergent ratio of 1:5–1:40, and it is characterized by a partial decrease in ThT fluorescence. The kinetic measurements showed that at these concentrations of Chobimalt, the elongation phase began after ∼40 min. For comparison, this time (∼40 min) is sufficient for insulin in the presence of 100 µM Chobimalt to complete fibrillization and reach the plateau phase. In addition, more curly and wavy fibrils were detected by AFM. For a better illustration of the curli morphology of these fibrils, AFM images of less diluted samples are shown in Figure 5.

Previously, Manno et al. observed that very long wavy-like insulin fibrils possess a tendency to form loops with a diameter of the order of half a micron after the application of a double quenching technique (Manno et al., 2007). Similarly, wavy fibrils and the formation of the coiled spring-like fibrils have been described for the tau-K18 domain under some aggregation conditions (Ramachandran and Udgaonkar, 2012). Ramachandran et al. suggest that these wavy fibrils could be fibrils that have become completely uncoiled (Ramachandran and Udgaonkar, 2012). Similar curled/wavy fibrils morphology has been observed for fibrils formed by full-length htau40 and three-repeat domain (K19) constructs (Wegmann et al., 2010). Highly curved fibrils were formed by bacterial protein MinE in the presence of 20 mM sucrose (Chiang et al., 2015). However, the fibril bending and formation of rings may also be induced by physical factors, as demonstrated by Jordens et al. (Jordens et al., 2014). The authors proposed that spontaneous curvature is governed by structural characteristics on the molecular level and is to be expected when a chiral and polar fibril are placed in an inhomogeneous environment such as an interface (Jordens et al., 2014). In our study, the morphology of fibrils changes correlates well with the differences observed in the kinetics of insulin fibrillization in the presence of Chobimalt. This supports our assumption of different conformational changes in insulin molecules after interaction with Chobimalt. We hypothesize that the different morphology of the formed insulin fibrils results from the gradual binding of Chobimalt to different binding sites on unfolded insulin. A similar explanation and the existence of such binding sites with different binding energies was shown previously for the nonionic detergents TX-100 and DDM (Siposova et al., 2019a; Siposova et al., 2021a). In addition, the published observation that several proteins may form polymorphic wavy and curled fibrils supports the assumption that the process of fibrillation is complex, and emphasizes the importance of protein partially-unfolded state which undergo the process of fibrils formation; i.e., certain experimental conditions, or the presence of “additives” may dramatically change not only kinetics but also the morphology of fibrillar aggregates (Ramachandran and Udgaonkar, 2012; Jordens et al., 2014; Chatani et al., 2015; Chiang et al., 2015).

### 3.3 Nanoscale structure examination using small-angle neutron scattering and small-angle X-ray scattering

**FIGURE 6:**
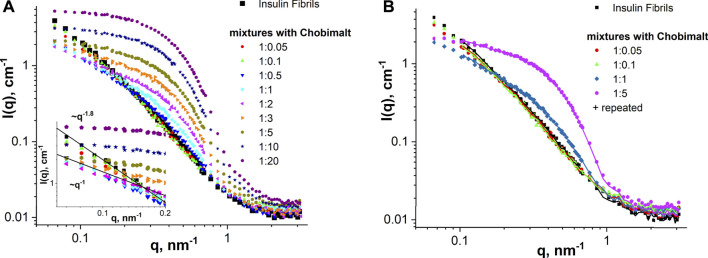
SANS analysis. **(A)** SANS curves of insulin fibrils formed alone and in the presence of different concentrations of Chobimalt. The inset graph represents the initial part of SANS curves for q below 0.2 nm^-1^ together with power-law lines I(q)∼q^-1^ and I(q)∼q^-1.8^. **(B)** SANS examination of the stability of insulin fibrils alone and formed in the presence of Chobimalt over time (symbols—initial samples and solid lines—samples measured after 1-week storage).

SANS and SAXS measurements were performed to obtain morphological information on insulin fibrils formed in presence of Chobimalt. Analysis of SANS data obtained in the study of insulin fibrils formed in the absence of Chobimalt *(control)* and in different concentrations of Chobimalt is presented in Figure 6A. The scattered intensity (SANS signal) of insulin amyloid fibrils formed alone and in presence of a low concentration of Chobimalt (1:0.05 and 1:0.1) indicate the scattering from elongated large-size aggregates [power-law behavior with I(q)∼q^-1.8^]. In concentration ratios, 1:0.5, 1:1, and 1:2 obtained SANS curves indicate the presence of cylinder-like objects (power-law behavior with I(q)∼q^-1^). Further increase of Chobimalt concentration (starting from the ratio of 1:5) caused the main SANS signals to come from Chobimalt micelles and overlay/disappear SANS signals from insulin fibrils. Important to note that SANS experiments were performed at a high concentration of insulin, but similarly to the ThT assay we have observed three different processes depending on the concentration of Chobimalt. It should be noted that during the measurements, the scattering of micelles is added to the scattering of amyloid structures. This is not interference, but a technical difficulty, which could have been solved, ideally, by minimizing the micelle contribution by using deuterated surfactants. However, these are not available for such novel molecules as Chobimalt. Nevertheless, the current data allow us to extract reliable information on the components of the two or more component solution. The power law behavior at small q is I(q)∼q^-1.6^ for pure amyloid fibrils *(control)* and mixtures with a low Chobimalt content, and it changes for I(q)∼q^-1^ for amyloid fibrils with intermediate content of Chobimalt. Since the Chobimalt alone forms micelles with a horizontal asymptote towards q = 0, the observed slope change can only be caused by amyloid aggregates, that are affected by some of the Chobimalt molecules. This is a direct indication of a change in the structural organization of protein aggregates. All these observations correlate well with other methods and confirm the different structures of amyloid aggregates.

This observation supports our recent suggestion that the ratio between the protein and detergent defines the outcome, not the total amount of detergent (Siposova et al., 2019a). Depending on this ratio, the effect of surfactant varies from minor changes in morphology to the formation of protein fibrils with sharply altered morphology.

By comparing experimental SANS data for the same samples measured within several days difference (Figure 6B), we can conclude almost full coincidence of SANS curves for all samples verified. Thus, it is a clear indication that the nanoscale structure of the samples remains constant at least during a 1-week timescale, i.e., there are no structural changes in the mixtures with time (during 7 days). The general behaviour of SANS curves obtained for Chobimalt solutions in D_2_O-DCl buffer corresponds well to an ellipsoid of revolution shape (polar radius is about 30 Å and equatorial radius is about 60 Å), which is typical for micelles of non-ionic detergents.

Therefore, obtained SANS results can be summarized as follows: SANS data fully support the existence of different stages of insulin fibrillization in the presence of different concentrations of Chobimalt (low, intermediate, and high). Even though at the highest concentration of Chobimalt, the SANS signals from insulin fibrils were partially overlaid by micelles signals, within these three concentrations regions, morphologically different fibrils were formed at least at the first and second concentrations regions, which was also confirmed by AFM. Additionally, the different morphologies were in correlation with distinct kinetics of fibrillization.

**FIGURE 7:**
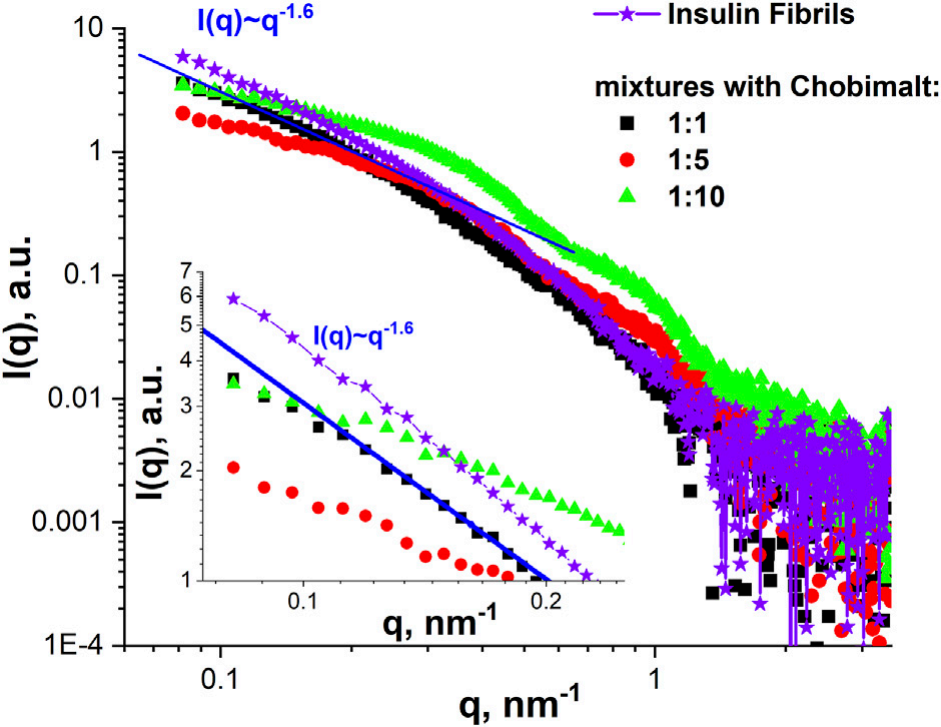
SAXS analysis. SAXS curves of insulin fibrils formed alone and in the presence of different concentrations of Chobimalt. The inset graph represents the initial part of SAXS curves for q below 0.25 nm^-1^ together with the power-law line I(q)∼q^-1.6^.

The SANS data were supported by SAXS experiments which were performed at selected concentrations of Chobimalt, *i.e*., the same tendency was observed in the SAXS curves for the initial insulin fibrils and mixtures with low content of Chobimalt, namely power-law behaviour with I(q)∼q^-1.6^ (Figure 7). With increasing Chobimalt concentration, the SAXS signal from fibrils decreased. Due to different scattering contrasts in SAXS and SANS experiments, SAXS signals from protein aggregates can be seen even for the highest amount of Chobimalt.

It should be mentioned, that the present results, showing that the increase of Chobimalt concentration leads to the changes in the morphology of protein fibrils, and the three stages of insulin fibrillization depending on the Chobimalt concentration, correlates well with our previous study of insulin and lysozyme amyloids fibrils under addition of fullerenes, in which the different stages of the disassembly process with respect to the size and morphology of the aggregates were observed by small-angle neutron scattering (Siposova et al., 2020). The unique properties of scattering methods are that they provide structural information at the nanoscale for each component in multicomponent solutions and the sensitivity of the method depends just on the scattering contrast between matrix and inhomogeneity, concentration and square of the volume of the particles. Also very recently quite similar concentration-dependent behavior was concluded by SAXS but for the globular protein β-lactoglobulin with an amphoteric surfactant, N,N-Dimethyldodecylamine N-oxide (Thompson et al., 2022). Furthermore, by molecular dynamics simulations of hen egg-white lysozyme (HEWL), it was found that the structural fluctuation of HEWL decreases as the concentration of C60 increases (Lee, et al., 2022). It can be concluded that the SANS and SAXS study, the CD, AFM, and ThT fluorescence data on the dose-dependent effect of Chobimalt on the formation of insulin fibrils correlate well with the results of previous works on the concentration-dependent effect of additives on the behavior of proteins and protein aggregates.

## 4 Conclusion

**FIGURE 8:**
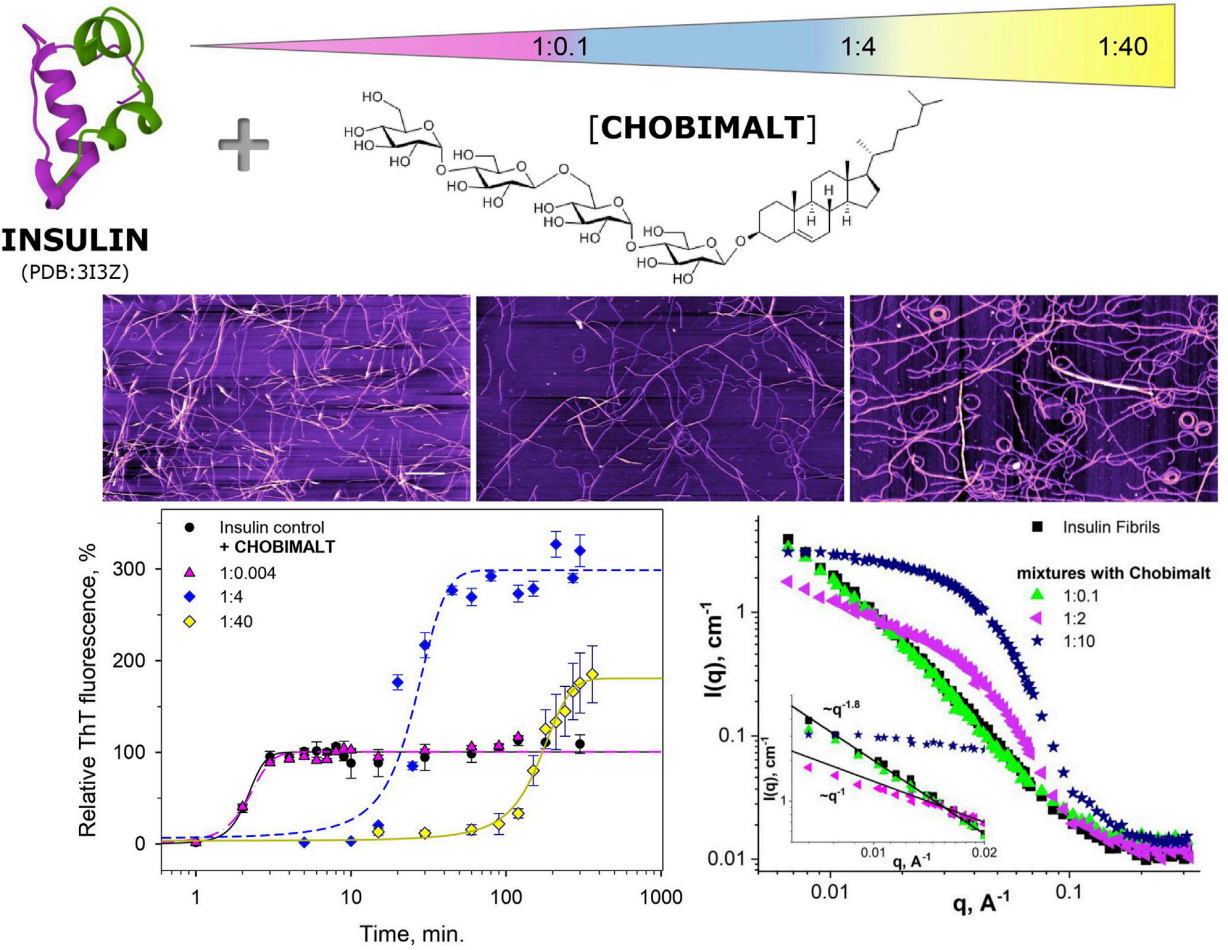
Schematic presentation of the effect of Chobimalt on insulin amyloid fibrillization.

It is well-known that the proteins amyloid fibril formation particularly the morphology of the formed fibrils (so-called polymorphism) highly depends on external conditions. In the present work, we have demonstrated a dose-dependent effect of cholesterol-based detergent, Chobimalt on the kinetic of insulin fibrillization and morphology of formed fibrils. Depending on the protein to Chobimalt molar ratio we observed the dramatically changed kinetics and morphology of fibrillar aggregates, as demonstrated in the schematic presentation (Figure 8). The fibrils appear to be more flexible and wavy-like with a tendency to form circles. It is likely that at low pH, elevated temperature and agitation the insulin molecules form a heterogeneous mix of unfolded intermediates, which subsequently generate amyloidogenic nuclei. We hypothesize that the different morphology of the formed insulin fibrils is the result of the gradual binding of Chobimalt to different binding sites on unfolded insulin. This hypothesis may be oversimplified since protein fibril aggregation is a complex process affected by various parameters. However, the similarity of the effect caused by Chobimalt with the effect triggered by Triton X-100 and dodecyl maltoside (Siposova et al., 2019a; Siposova et al., 2021a) indicates similarity in the mechanism. While further investigations are essential to clarify different aspects of Chobimalt-insulin interaction, we believe that our results are important for the understanding of the possible role of cholesterol in amyloid fibrils formation.

In conclusion, our data support the hypothesis that the interplay between cholesterol and insulin is an important feature of amyloidogenesis (for review see Gamba et al., 2019).

## Data Availability

The original contributions presented in the study are included in the article/supplementary material, further inquiries can be directed to the corresponding authors.
